# Prebiotic and synbiotic effect on rumen papilla length development and rumen pH in 12-week-old calves

**DOI:** 10.14202/vetworld.2021.2883-2888

**Published:** 2021-11-11

**Authors:** A. Arne, A. Ilgaza

**Affiliations:** Department of Anatomy and Physiology, Preclinical Institute, Faculty of Veterinary Medicine, Latvia University of Life Sciences and Technologies, Jelgava, Latvia

**Keywords:** calf, pH, prebiotic, rumen, symbiotic

## Abstract

**Background and Aim:**

Europe and the USA have banned antibiotics use as growth promoters. There is a need for alternative products that can ensure production and health protection. Prebiotics has been proposed as alternatives because these materials have wide-ranging physiological effects on gut function, activity of the large intestinal microflora, mineral absorption, and immunity. The aim of this study was to determine the effect of three different doses of inulin, a prebiotic, in combination with probiotic *Enterococcus faecium* (a new synbiotic) on postnatal rumen development by comparing rumen papilla length, width, muscle layer thickness, and content pH level.

**Materials and Methods:**

Randomly selected 23 (±5)-days-old healthy male Holstein crossbreed calves, weighing 50 kg (±5 kg), were randomly allocated to seven groups, ten in each group. The calves were kept in a pen of 5, under the same conditions and were fed twice a day, ~3.5 liters of whole milk per feeding. Control group (C n=10) was fed with whole milk only (no additives were added). The six other groups (three prebiotics and three synbiotics) received food additives with their morning milk feeding. The source of prebiotics, *Jerusalem artichoke* powder concentrate (JAPC) contained 50% of inulin. JAPC in doses of 6 g, 12 g, or 24 g were added to the milk. Formed prebiotic groups were denoted as PreG6, PreG12, and PreG24. To evaluate if the addition of the probiotic *E. faecium* 2×10^9^ colony forming unit g^−1^ to manufacturer recommended dose of 0.25 g improves inulin effect on rumen, it was added to all their JAPC doses. The new content synbiotic groups were denoted as SynG6, SynG12, and SynG24. On day 57 of the study, when all calves were approximately 12 weeks old, they were slaughtered in a certified slaughterhouse. Tissue cultures for histological analysis were obtained from *Saccus dorsalis* and *Saccus ventralis* of the rumen. Tissue culture staining for histology was carried out using hematoxylin and eosin staining method. Rumen histological samples were used to measure papilla length, width, and muscle layer thickness. Each sample was used to make five measurements on the present rumen papilla.

**Results:**

The results showed that by adding 12 g of inulin to whole milk when feeding calves improves rumen papilla development, which is seen by increased length and width of papilla, especially in the *Saccus ventralis* region. By combing this dose of inulin with 0.25 g of *E. faecium*, a significant increase of papilla is achieved. *Saccus ventralis* muscle layer in the rumen is thicker than it is in *Saccus dorsalis* regardless of addition of prebiotics or synbiotics.

**Conclusion:**

The addition of inulin to whole milk can influence the pH of the rumen by making it more alkaline. The addition of prebiotic inulin and a novel synbiotic (inulin combined with *E. faecium*) can accelerate postnatal rumen development and improve its functionality.

## Introduction

The goal of each cattle rancher and veterinarian is to have a healthy and highly productive cattle herd. To reach this goal, it is important to take care of cattle health from their birth, because only then high-quality dairy cows and beef cattle can be achieved. The number one cause of premature death and illness in calves after birth is diarrhea [1]. Gastrointestinal tract health issue prevention is an important development factor for healthy young cattle [2]. Up until recently, to prevent illness and to promote calf growth, antibiotics were added to calf food sources. However, this addition of antibiotics to food promotes antibiotic-resistant bacteria development [2], so since 2006 European Union has banned the use of antibiotics additives to food for illness prevention purposes [3]. There has been a need for new alternative food additive research to improve animal health and growth [4].

Studies show that prebiotics and probiotics and their combinations (synbiotics) have a positive effect on calf health and growth [2,5-7]. Prebiotics are oligosaccharides that cannot be digested by digestive enzymes; they can be used by gut microorganisms to speed their growth and development. They stimulate beneficial bacteria growth and activity by protecting the intestinal walls from pathogens and reducing pathogen proliferation in gastrointestinal tract [8,9]. Probiotics are live beneficial microorganisms that protect gastrointestinal tract walls from pathogens, improve its barrier function, stimulate bactericide production and by modeling the immune system, stimulate local and overall intestine immunity [10-12].

The aim of this study was to determine the effect of three different doses of inulin, a prebiotic, in combination with probiotic *Enterococcus faecium* (a new synbiotic) on postnatal rumen development by comparing rumen papilla length, width, muscle layer thickness, and content pH level.

## Materials and Methods

### Ethical approval

All procedures performed in the present study were in accordance with the ethical standards. Research Committee of the Faculty of Veterinary Medicine, Latvia University of Life Sciences and Technologies approved this study (protocol no. 2017/2).

### Study period and location

The study was carried out from May 2013 to June 2016. The study was carried out at 420 cow dairy farms in Latvia. Laboratory analyses were carried out at Faculty of Veterinary Medicine, Latvia University of Agriculture.

### Animals

The research was carried out on 420 cow dairy farms in Latvia. Randomly selected 23 (±5)-days-old healthy male Holstein crossbreed calves, weighing 50 kg (±5 kg), were randomly allocated to seven groups, ten in each group. The calves were kept in a pen of 5, under the same conditions and were fed twice a day, ~3.5 liters of whole milk per feeding. Water and hay were freely available 24 h/day, and fodder was added 2 weeks after the start of the study when the animals were 6 weeks old.

### Experimental design

Seven study groups were formed by including ten calves in each group. Control group (C, n=10) was fed with whole milk only (no additives were added). The six other groups (three prebiotics and three synbiotics) received food additives with their morning milk feeding. The source of prebiotics, *Jerusalem artichoke* powder concentrate (JAPC) contained 50% of inulin [13]. JAPC in doses of 6 g, 12 g, or 24 g were added to the milk. Formed prebiotic groups were denoted as PreG6, PreG12, and PreG24. To evaluate if the addition of the probiotic *E. faecium* 2×10^9^ colony forming unit (CFU) g^−1^ to manufacturer recommended (Protexin International Ltd., South Petherton, UK) dose of 0.25 g improves inulin effect on rumen, it was added to all their JAPC doses. The new content synbiotic groups were denoted as SynG6, SynG12, and SynG24.

Feeding with food additives was carried out for 56 days while monitoring the overall health and weight gain of the animals [14]. On day 57 of the study, when all calves were approximately 12 weeks old, they were slaughtered in a certified slaughterhouse. During slaughter, the gastrointestinal tract organs were examined and samples were obtained on site. In the rumen opening, an incision was made to obtain intraruminal pH of *atrium ruminis* using a digital pH meter (pH 3310 SET, WTW, Poland).

Tissue cultures for histological analysis were obtained from *Saccus dorsalis* and *Saccus ventralis* of the rumen. Samples (0.5×0.7cm) were washed with 0.9% NaCl solution and placed in 10% formalin for at least 48 h. Sample dehydration and preparation using the standard method to encapsulate in paraffin blocks a tissue processor (Tissue-Tek II) was used. Tissue culture staining for histology was carried out using hematoxylin and eosin staining method [15].

Rumen histological samples were used to measure papilla length, width, and muscle layer thickness. Papilla’s length measurements were taken from papilla’s epithelial apical end to the mucosal membrane of the muscle plate; papilla width measurements were made perpendicular to the length measurements in the center of the papillae. Each sample was used to make 5 measurements on the present rumen papilla [16].

### Statistical analysis

The assumption of normal data distribution was assessed by Shapiro-Wilk’s test and visual inspection of their histograms and normal Q-Q plots. The assumption of homogeneity of variances was tested by Levene’s test. Significance was tested by applying the student t-test. Values of less than 0.05 (p<0.05) were considered significant.

## Results

Obtained results suggest that the prebiotic inulin containing JAPC and novel synbiotic addition to fodder impacts the development of rumen in calves. Even though papilla length, width, and muscle layer comparison of *Saccus ventralis* and *Saccus dorsalis* in the rumen of 12 week old calf groups after day 56 of the study cannot be seen as universality significant, as rumen papilla lengths data was scattered (Table-1). However, detailed statistical analysis showed that there are significant differences between experimental groups.

**Table-1:** Rumen *Saccus ventralis* papilla length, width, and muscle layer thickness in 12-week-old calves.

Group	*Saccus ventralis* (x̄ µm±SD)		

	Papilla length	Papilla width	Muscle layer
C	831.6±485.77	149.7±55.63	383.6±142.11
PreG6	818.3±415.85	163.38±47.27*	363.4±108.98
PreG12	831.4±485.05	163.29±45.01	427.1±136.89
PreG24	937.8±300.98	173.29±67.90**	407.1±106.51
SynG6	969.0±479.69*	180.2±45.81**	302.1±88.24*
SynG12	1010.7±479.28**	183.3±47.85**	516.4±110.43**
SynG24	1067.1±570.92**	192.26±49.30**	399.1±106.83

** p<0.01 compared to CoG;

* p<0.05 compared to CoG

Out of all experimental prebiotic groups, the longest rumen *Saccus ventralis* papilla’s were observed in calves of PreG24, shortest in PreG6 group; however, by adding the medium dose of prebiotics (PreG12) papilla length was higher than groups in C and PreG6; however, this is not deemed to be a significant difference when comparing to C (Table-1). Comparison of rumen *Saccus ventralis* papilla length in C to synbiotic experimental groups showed that C has significantly (p<0.01) shorter papilla than SynG12 and SynG24 group calves; comparison of C to SynG6 showed a significant difference (p<0.05) in length comparison as well. Group SynG24 showed the longest papilla as they were fed the highest dose of inulin and *E. faecium*.

By analyzing the width of *Saccus ventralis* papilla, it was determined that *Saccus ventralis* papilla in control group C is significantly more narrow than in experimental group PreG6 (p<0.05) and PreG24 (p<0.001). Even though group PreG12 showed wider papilla than control group C, the difference was not deemed to be significant (Table-1). Synbiotic addition to fodder improved papilla width growth. All synbiotic group calves *Saccus ventralis* papilla width were significantly (p<0.001) wider than the control group. The widest papilla was observed in the group that was fed the highest dose of inulin with *E. faecium*, SynG24 (Table-1).

By analyzing rumen papillae in *Saccus dorsalis* region, we found that by probiotic inulin uptake, the longest papillae were observed in PreG12 group calves. Analyzing papillae length and comparing them to control group, no significance was found in papillae length differences (Table-2). All synbiotic experimental group papillae were the shortest when compared to *Saccus dorsalis* in control group calves. Statistically significantly (p<0.001) shorter they were for groups SynG12 and SynG24, however between groups SynG6 and C, no differences of statistical significance were found (Table-2).

**Table-2:** Rumen *Saccus dorsalis* papilla length, width, muscle layer thickness, and intraruminal pH measurements in 12-week-old calves.

Group	*Saccus dorsalis* (x̄ μm±SD)			pH (x̄±SD)

	Papilla length	Papilla width	Muscle layer	
C	301.16±62.21	199.2±59.29	329.4±74.71	5.8±0.60
PreG6	303.2±52.65	192.7±51.98	336.0±154.49	5.8±0.61
PreG12	313.6±67.51*	218.9±68.24*	285.6±55.64**	6.1±0.38
PreG24	302.5±84.47	208.66±80.71	295.3±81.20**	6.2±0.58
SynG6	302.7±67.38	222.9±56.93**	210.2±60.19**	6.1±0.73
SynG12	329.6±60.02**	199.4±52.88	327.6±166.62*	6.1±0.38
SynG24	360.41±61.95**	200.9±47.75	331.4±96.26*	6.0±0.24

** p<0.01 compared to CoG;

* p<0.05 compared to CoG

The analysis of rumen *Saccus dorsalis* papillae width shows that the widest papillae were found in PreG12 group calves. They are significantly wider than in C (p<0.05) and PreG6 (p<0.01) group calves.

By comparing mean papillae width in synbiotic experimental group animals, it was found that group SynG12 had the narrowest papillae in this study. Comparison among width, the mean in synbiotic groups shows that group SynG6 has significantly (p<0.001) wider *Saccus dorsalis* papillae than SynG12 and significantly (p<0.01) wider than SynG24 group calves (Table-2).

Analysis of mean muscle layer thickness in *Saccus ventralis* data (Table-1) shows that control group C animals show no statistical difference between prebiotic experimental group animals. The thickest muscle layer in *Saccus ventralis* between synbiotic group calves was found in SynG12 group calves, but the thinnest for SynG6 group animals (Table-1). Group SynG6 animal muscle layer thickness is significantly (p<0.01) more narrow than in group SynG12. Group SynG6 animals have significantly (p<0.01) more narrow *Saccus ventralis* muscle layer thickness than groups SynG12 and SynG24 (Table-1).

Muscle layer in rumen *Saccus dorsalis* (Table-2) for groups PreG12 (p<0.01) and PreG24 (p<0.05) is significantly thicker when compared to C group. Comparing C to SynG24, group SynG24 animals have a significantly thicker muscle layer than C group.

By analyzing muscle layer thickness measurement data, we observed that all group animals have a thicker muscle layer in *Saccus ventralis* than in *Saccus dorsalis*. Moreover, the differences between *Saccus dorsalis* and *Saccus ventralis* muscle layer thickness measurements were significant for groups C, PreG12, and PreG24.

To evaluate if the addition of 0.25 g of probiotic *E. faecium* 2×10^9^ CFU g^−1^ improves inulin effect on the rumen, papillae sizes of both rumen parts were compared. By comparing rumen *Saccus ventralis*, the most efficient prebiotic PreG24 group to the most efficient synbiotic SynG24 group, it was found that SynG24 group calves have significantly (p<0.01) longer and significantly (p<0.01) wider papillae than SynG24 group calves. For rumen *Saccus dorsalis*, the longest and widest papillae were observed in group PreG12 animals. Comparing these results to SynG12 group animals, no statistical differences were found. No differences were found in muscle layer thickness measurement data between *Saccus dorsalis* and *Saccus ventralis* regions. This suggests that the addition of 0.25 g of *E. faecium* to 12 g of inulin significantly improves papillae sizes in rumen *Saccus ventralis* region.

Rumen pH influences its microbiome functionality status. The most optimal rumen environment was found in group C, followed by PreG6. Less acidic rumen content pH was found for groups PreG12 and PreG24 exceeding 6 pH units (Table-2). No significant differences were found between the pH measurements of the rumen content of the C and the rest of the experimental groups.

However, there were differences found among the experimental groups; PreG6 group animal rumen pH content was found to be significantly (p<0.05) more acidic than PreG12 group animal rumen. The pH of all synbiotic experimental group, animal rumens exceeds 6 pH units (Table-2). Comparing all possible mean pH measurements among the groups, no statistical differences were found.

## Discussion

The mucosal lining of the rumen is made out of numerous papillae, which are the main absorbents of nutrients and water that are needed for proper growth and development [17], which leads up to suggest that the longer and wider the papillae, the larger the surface area for absorption and nutrients can be processed more effectively. The size of the papillae is the main indicator that can serve as the gold standard method to determine the effect of nutrient addition to fodder on rumen development [18]. It is known that the rumen epithelia is critical for short chain fatty acid absorption [19,20].

Studies show that fodder significantly impacts papillae size. Górka *et al*. [21] suggest that calves who are fed whole milk have larger papillae than those who are fed a milk substitute. Studies prove that animals that are fed roughage in addition to hay have faster rumen development that facilitates a process that will ultimately affect the mass of rumen, its volume, as well as muscle layer thickness than those who are only fed hay [22,23]. Costa *et al*. [24] suggest that there is no difference between feeding roughage or not on papillae length; however, authors suggest that addition of manno-oligosaccharides (MOS) prebiotic (similar to our used inulin) to milk has a positive effect on developing longer papillae. This can suggest that prebiotics can stimulate food breakdown processes in the rumen that improve papillae development. Many studies that used the fermentation products of *Saccharomyces cerevisiae*, as well as prebiotics (glycans and oligosaccharides) showed improved rumen papillae length and width development [18,25,26]. One explanation for this phenomenon is that prebiotics improves formation of soluble fatty acids because the final product that is formed by breaking down prebiotics are soluble fatty acids [27], which can further suggest it can have an impact on the following breakdown enzyme activity in the intestines. Roughage trains the muscle of the rumen and improves its development; however, the thickness of its muscle layer is not dependent on the development of the rumen epithelia.

Our study suggests the addition of prebiotics, inulin specifically, to food can significantly improve rumen papillae development and muscle layer thickness. We observed improved growth of *Saccus ventralis* muscle and longer *Saccus ventralis* papillae in those calves that were fed medium and high doses of inulin and its combination with *E. faecium*. Inulin has improved the development of these tissues as the rumen requires a longer breakdown process for roughage; however, this suggestion is not conclusive.

Good feeding practice suggests that the optimal pH of rumen for complex food breakdown should be between 5.8 and 6.4 pH units in order for the optimal food breakdown process [28]. Prebiotics play a huge role in the absorption and transport of nutrients as well as increasing soluble fatty acid forming and nutrient breakdown that increases the formation of butyrate, which serves as an energy source for intestine epithelial cells and increases absorption [29]. A study that used polysaccharide beta-glucan as its source of prebiotics showed increased pH in the rumen and better food breakdown [30]. Studying ruminants, it is important to understand the changes in rumen pH content measurements where primary food breakdown and fermentation takes place that can significantly impact the overall health of growing cattle. In our study, C group had the most acidic rumen environment but less acidic (pH 6.45±0.38) in PreG12 group animals. Results suggest that calves that received the lowest dose of inulin (3 g/day), the rumen pH content level is similar to the control group (average of 5.7 pH). Following the obtained data, we can conclude that 3 g/day dose of inulin cannot achieve the optimal literature suggested rumen pH content level. Animals that were fed double the amount (6 g/day) of inulin reported significantly (p<0.05) higher pH levels (pH 6.1±0.39) than animals that received a dose 3 g/day of inulin. This pH level provides the optimal pH that the literate suggests for the optimal breakdown of fiber and starch [28]. The sum of our study coincides with the outcome obtained by Senevirathne *et al.*, [31], where their results of studying oligosaccharides for calves, no significant differences were found in the pH level of the digestive tract between control and experimental group’s animals. Król [8] fed 2 g of MOS prebiotics a day and achieved a higher rumen pH content than the control group, but even more significant (p<0.01) difference was achieved in the rumen of calves that were fed 4 g of MOS compared to the control group.

Groups that received the novel synbiotic with milk showed an intraruminal pH level of 6.01±0.45, which was found to be higher than in the control group. This coincides with Núñez-Benítez *et al*. [32] study where Holstein type bull was fed synbiotics that contained inulin and yeast and showed lower rumen pH content level than control groups.

## Conclusion

It can be concluded that adding 12 g of inulin to whole milk when feeding calves improves rumen papillae development, which is seen by increased length and width of papillae, especially in the *Saccus ventralis* region. By combining this dose of inulin with 0.25 g of *E. faecium*, a significant increase of papillae length and width was achieved. Muscle layer in the rumen *Saccus ventralis* is thicker than it is in *Saccus dorsalis* regardless of addition of prebiotics or synbiotics. The addition of inulin to whole milk can influence the pH of the rumen by making it more basic. The addition of prebiotic inulin and a novel synbiotic (inulin combined with *E. faecium*) can accelerate postnatal rumen development and improve its functionality; however, more studies are needed.

## Authors’ Contributions

AA: Performed the study, data analysis, and drafted the manuscript. AI: Performed data analysis, drafted, and revised the manuscript. Both authors read and approved the final manuscript.

## References

[1] Cangiano L.R, Yohe T.T, Steele M, Renaud D.L (2020). Invited review:Strategic use of microbial-based probiotics and prebiotics in dairy calf rearing. Appl. Anim. Sci.

[2] Hasunuma T, Kawashima K, Nakayama H, Murakami T, Kanagawa H, Ishii T, Akiyama T, Yasuda K, Terada F, Kushibiki S (2011). Effect of cellooligosaccharide or synbiotic feeding on growth performance, fecal condition un hormone concentrations in Holstein calves. Anim. Sci. J.

[3] Geigerová M, Bunešová V, Vlková E, Salmonová H, Rada V (2017). Selection of prebiotic oligosaccharides suitable for synbiotic use in calves. Anim. Feed Sci. Technol.

[4] Kertz A.F, Hill T.M, Quigley J.D, Heinrichs A.J, Linn J.G, Drackley J.K (2017). 100-year review:Calf nutrition and management. Int. J. Dairy Sci.

[5] Agazzi A, Tirloni E, Stella S, Maroccolo S, Ripamonti B, Bersani C, Savoini G (2014). Effects of species-specific probiotic addition to milk replacer on calf health and performance during the first month of life. Ann. Anim. Sci.

[6] Le O.T, Dart P.J, Harper K, Zhang D, Schofield B, Callaghan M.J, McNeill D.M (2017). Effect of probiotic *Bacillus amyloliquefaciens* strain H57 on productivity and the incidence of diarrhoea in dairy calves. Anim. Prod. Sci.

[7] Jonova S, Ilgaza A, Zolovs M (2021). The impact of inulin and a novel synbiotic (*yeast Saccharomyces cerevisiae* strain 1026 and inulin) on the development and functional state of the gastrointestinal canal of calves. Vet. Med. Int.

[8] Król B (2011). Mannon-oligosaccharides, inulin and yeast nucleotides added to calf milk replacers on rumen microflora, level of serum immunoglobulin and health condition of calves. Electron. J. Pol. Agric. Univ.

[9] Markowiak P, Śliżewska K (2017). Effects of probiotics, prebiotics, and synbiotics on human health. Nutrients.

[10] Bidarkar V.K, Swain P.S, Ray S, Dominic G (2014). Probiotics:Potential alternative to antibiotics in ruminant feeding. Trends Vet. Anim. Sci.

[11] Hill C, Guarner F, Reid G, Gibson G.R, Merenstein D.J, Pot B (2014). Expert consensus document. The international scientific association for probiotics and prebiotics consensus statement on the scope and appropriate use of the term probiotic. Nat. Rev. Gastroenterol. Hepatol.

[12] Gibson G.R, Hutkins H, Sanders M.E, Prescott S.L, Reimer R.A, Salminen S.J, Scott K, Stanton C.K, Swanson S, Cani P.D, Verbeke K, Reid G (2017). Expert consensus document:The international scientific association for probiotics and prebiotics (ISAPP) consensus statement on the definition and scope of prebiotics. Nat. Rev. Gastroenterol. Hepatol.

[13] Arne A, Ilgaza A (2021). The effect of synbiotic inulin and enterococcus bacteria on digestive health and weight gain in calves. Agron. Res.

[14] Ārne A, Ilgaža A (2016). Different dose inulin feeding effect on calf digestion canal state and development. Res. Rural Dev.

[15] Carson F.L, Hladik C (2009). Histotechnology:A Self-Instructional Text.

[16] Gelsinger S.L, Coblentz W.K, Zanton G.I, Ogden R.K, Akins M.S (2020). Physiological effects of starter-induced ruminal acidosis in calves before, during, and after weaning. Int. J. Dairy Sci.

[17] Graham C, Simmons N.L (2005). Functional organization of the bovine rumen epithelium. Am. J. Physiol. Regul. Integr. Comp. Physiol.

[18] Lesmeister K.E, Tozer P.R, Heinrichs A.J (2004). Development and analysis of a rumen tissue sampling procedure. Int. J. Dairy Sci.

[19] Kramer T, Michelberger T, Gürtler H, Gäbel G (1996). Absorption of short-chain fatty acids across ruminal epithelium of sheep. J. Comp. Physiol. B.

[20] Penner G.B, Aschenbach J.R, Gabel G, Rackwitz R, Oba M (2009). Epithelial capacity for apical uptake of short chain fatty acids is a key determinant for intraruminal pH and the susceptibility to subacute ruminal acidosis in sheep. Nutr. J.

[21] Górka P, Kowalski Z.M, Pietrzak P, Kotunia A, Jagusiak W, Zabielski R (2011). Is rumen development in newborn calves affected by different liquid feeds and small intestine development?. Int. J. Dairy Sci.

[22] Baldwin R.L, Mc Leod K.R, Klotz J.L, Heitmann R.N (2004). Rumen development, intestinal growth and hepatic metabolism in the pre-and postweaning ruminant. Int. J. Dairy Sci.

[23] Khan M.A, Weary D.M, von Keyserlingk M.A.G (2011). Hay intake improves performance and rumen development of calves fed higher quantities of milk. Int. J. Dairy Sci.

[24] Costa N.A, Pansani A.P, Henrique de Castro C, Basile Colugnati D, Xaxier C.H, Guimarães K.C, Rabelo L.A, Nunes-Souza V, Fernuno Caixeta L.S, Ferreira R.N (2019). Milk restriction or oligosaccharide supplementation in calves improves compensatory gain un digestive tract development without changing hormone levels. PLoS One.

[25] Brewer M.T, Anderson K.L, Yoon I, Scott M.F, Carlson S.A (2014). Amelioration of salmonellosis in pre-weaned dairy calves fed *Saccharomyces cerevisiae* fermentation products in feed and milk replacer. Vet. Microbiol.

[26] Xiao J.X, Alugongo G.M, Chung R, Dong S.Z, Li S.L, Yoon I, Cao Z.J (2016). Effects of *Saccharomyces cerevisiae* fermentation products on dairy calves:Ruminal fermentation, gastrointestinal morphology, and microbial community. Int. J. Dairy Sci.

[27] Kaufhold J, Hammon H.M, Blum J.W (2000). Fructooligosaccharide supplementation:Effects on metabolic, endocrine and hematological traits in veal calves. J. Vet. Med. A Physiol. Pathol. Clin. Med.

[28] Highfill G, Lalman D (2005). The ruminant animal. Nat. Cattle.

[29] Singh A.K, Kerketta S, Yogi R, Kumar A, Ojha L (2017). Prebiotics:The new feed supplement for dairy calf. Int. J. Livest. Res.

[30] Kim H.S, Hong J.T, Kim Y, Han S.B (2011). Stimulatory effect of b-glucans on immune cells. Immune Netw..

[31] Senevirathne N.D, Anderson J.L, Metzger L (2019). Growth performance, nutrient utilization, and health of dairy calves supplemented with condensed whey solubles. Int. J. Dairy Sci.

[32] Núñez-Benítez V.H, Barreras A, Estrada-Angulo A, Castro-Pérez B.I, Urías-Estrada J.D, Zinn R.A, Plascencia A (2020). Evaluation of a standardized mixture of synbiotic-glyconutrients as a feed additive in steers fed a finishing diet:Site and extent of digestion, ruminal fermentation, and microbial protein synthesis. Livest. Sci.

